# Altered Functions of Neutrophils in Two Chinese Patients With Severe Congenital Neutropenia Type 4 Caused by *G6PC3* Mutations

**DOI:** 10.3389/fimmu.2021.699743

**Published:** 2021-07-08

**Authors:** Rongxin Dai, Ge Lv, Wenyan Li, Wenjing Tang, Junjie Chen, Qiao Liu, Lu Yang, Min Zhang, Zhirui Tian, Lina Zhou, Xin Yan, Yating Wang, Yuan Ding, Yunfei An, Zhiyong Zhang, Xuemei Tang, Xiaodong Zhao

**Affiliations:** ^1^ Department of Rheumatology & Immunology, Children's Hospital of Chongqing Medical University, Chongqing, China; ^2^ National Clinical Research Center for Child Health and Disorders, Children’s Hospital of Chongqing Medical University, Chongqing, China; ^3^ Ministry of Education Key Laboratory of Child Development and Disorders, Children’s Hospital of Chongqing Medical University, Chongqing, China; ^4^ Chongqing Key Laboratory of Child Infection and Immunity, Children’s Hospital of Chongqing Medical University, Chongqing, China

**Keywords:** G6PC3, neutrophils, severe congenital neutropenia, NETs, G-CSF treatment, apoptosis

## Abstract

**Background:**

SCN4 is an autosomal recessive disease caused by mutations in the *G6PC3* gene. The clinical, molecular, and immunological features; function of neutrophils; and prognosis of patients with SCN4 have not been fully elucidated.

**Methods:**

Two Chinese pediatric patients with *G6PC3* mutations were enrolled in this study. Clinical data, genetic and immunologic characteristics, and neutrophil function were evaluated in patients and controls before and after granulocyte colony-stimulating factor (G-CSF) treatment.

**Results:**

Both patients had histories of pneumonia, inguinal hernia, cryptorchidism, and recurrent oral ulcers. Patient 1 also had asthma and otitis media, and patient 2 presented with prominent ectatic superficial veins and inflammatory bowel disease. DNA sequencing demonstrated that both patients harbored heterozygous *G6PC3* gene mutations. Spontaneous and FAS-induced neutrophil apoptosis were significantly increased in patients, and improved only slightly after G-CSF treatment, while neutrophil respiratory burst and neutrophil extracellular traps production remained impaired in patients after G-CSF treatment.

**Conclusion:**

G-CSF treatment is insufficient for patients with SCN4 patients, who remain at risk of infection. Where possible, regular G-CSF treatment, long-term prevention of infection, are the optimal methods for cure of SCN4 patients. It is important to monitor closely for signs of leukemia in SCN4 patients. Once leukemia occurs in SCN4 patients, hematopoietic stem cell transplantation is the most important choice of treatment.

## Altered Functions of Neutrophils in Two Chinese Patients With Severe Congenital Neutropenia Type 4 Caused by *G6PC3* Mutations

Neutrophils are not only the most abundant innate effector cells of the human immune system, but the key to first line of immune defense against invading pathogens. A variety of antimicrobial functions, including generation of reactive oxygen species (ROS), phagocytosis of pathogens, degranulation, cytokine production, and generation of neutrophil extracellular traps (NETs), are executed by neutrophils (1–3).

Severe congenital neutropenia (SCN) is a primary immunodeficiency disease affecting the numbers and functions of neutrophils. The incidence of SCN is approximately six per million births. SCN type 4 (SCN4, MIM 612541) is an autosomal recessive disease caused by mutations in gene

*G6PC3*, which maps to chromosome 17q21.31, consists of six exons and encodes catalytic subunit 3 of glucose-6-phosphatase-β (4, 5). Glucose-6-phosphatase (G6P) is an enzyme that localizes in the endoplasmic reticulum (ER) and catalyzes the hydrolysis of G6P to glucose and inorganic phosphate. Neutrophils are capable of endogenous glucose production in their ER through the hydrolysis of G6P (6). In humans, there are three differentially expressed glucose-6-phosphatase catabolic genes: *G6PC1*, *G6PC2*, and *G6PC3*. The glucose-6-phosphatase-β protein (G6PC3) is ubiquitously expressed and anchored in ER by nine transmembrane helices that retain the active site within the ER lumen (7). G6PC3 is essential for neutrophil survival, energy homeostasis, and function properly (8), and G6PC3-deficient neutrophils are unable to hydrolyze endoluminal G6P to glucose, leading to impaired energy homeostasis, which manifests as ER stress, an enhanced rate of apoptosis, and impairments in respiratory burst, chemotaxis, calcium mobilization, and phagocytosis (6, 9, 10).

The major phenotype of SCN4 patients with *G6PC3* deficiency is severe peripheral blood neutropenia, and almost two thirds of SCN4 patients demonstrate intermittent thrombocytopenia (11). Further, some patients present with severe lymphopenia and thymic hypoplasia. Bone marrow examination may show maturation arrest in the myeloid lineage, or hyper- or normo-cellular bone marrow (12). Almost all patients present with recurrent infections, including sinopulmonary infections, otitis media, urinary tract infections, skin abscesses, and sepsis. Some patients also present with oral ulcers, periodontitis, stomatitis, gingivitis, and fungal infections. In addition, a prominent superficial venous pattern, congenital cardiac anomaly, inflammatory bowel disease (IBD), renal system malformation, genital anomalies (such as cryptorchidism), inguinal hernia, minor facial dysmorphism, minor skeletal and integument anomalies, pulmonary hypertension, endocrine abnormalities, intrauterine growth retardation, failure to thrive, and poor postnatal growth, as well as intellectual deficits, have also been reported (12). G6PC3-deficient neutrophils exhibit dysfunction, characterized by impairment of respiratory burst, chemotaxis, and calcium flux activities (8). Peripheral blood neutrophils isolated from patients with G6PC3 deficiency also showed increased apoptosis *in vitro* (5, 6, 13).

Granulocyte colony-stimulating factor (G-CSF) is used to treat neutropenia in patients with G6PC3 deficiency (5). But the effects of G-CSF treatment on G6PC3-deficient neutrophil functions, such as respiratory burst, pathogen phagocytosis, and generation of NETs remain unclear. To date, fewer than 100 cases of patients harboring *G6PC3* mutations have been reported in the literature. Consequently, the clinical, molecular, and immunological features; function of neutrophils; and prognosis of this disease have not been fully elucidated.

Here, we report characterization of two SCN4 patients with G6PC3 deficiency by analysis of their clinical, genetic, and immunological characteristics. Both patients received G-CSF treatment following diagnosis. After G-CSF treatment for 4 years, the parents of patient 1 chose to stop G-CSF treatment. We examined surface CXCR4 expression, respiratory burst, apoptosis, and generation of NETs in both patients before and during G-CSF treatment and after discontinuation of G-CSF treatment to provide information useful in the assessment of prognosis in SCN4 patients.

## Methods

### Patients and Ethics Statement

Two boys from two unrelated Chinese families were included in this study. At least one healthy control (HC) was included for comparison in each experiment. Whole blood samples were acquired from patients, patient family members, and age-matched healthy volunteers. Both patients with SCN4 were diagnosed by targeted gene panel sequencing, and the diagnoses were confirmed by Sanger sequencing. The study was conducted according to the Declaration of Helsinki. All study participants and HCs, or their guardians, provided written informed consent to participate in the study, which was approved by the Ethics Committee of the Children’s Hospital of ChongQing Medical University.

### Isolation of Human Granulocytes

Heparinized peripheral blood samples were obtained from patients and HCs. Granulocytes were isolated by dextran sedimentation and hypotonic lysis of residual red blood cells, and resuspended in Krebs Ringer phosphate buffer (KRG, pH 7.3: 120 mM NaCl, 5 mM KCl, 1.7 mM KH_2_PO_4_, 8.3 mM NaH_2_PO_4_, and 10 mM glucose). Briefly, after dextran sedimentation at 400 g, suspensions were centrifuged on Ficoll-Paque; plasma, mononuclear cells, and Ficoll fluid were removed; and the remaining erythrocytes were lysed by hypotonic treatment. Granulocytes were then washed in KRG buffer, resuspended in KRG supplemented with 1 mM Ca^2+^, and kept on melting ice until used.

### Flow Cytometry

Peripheral blood mononuclear cells (PBMCs) were isolated from freshly drawn heparin-treated blood of patients by means of the Ficoll density gradient centrifugation. PBMCs were stained with monoclonal antibodies (CD3, CD4, CD8, CD56, CD19, CD184; BD Biosciences, San Jose, CA, USA), and erythrocytes in the samples were lysed by incubation with lysing solution. Granulocytes were stained with mouse anti-human CD184, CD16b, or mAbs against human CD45, CD11b, CD16 (BD Biosciences, PharMingen). Cells were stained with conjugated monoclonal antibodies and isotype controls using standard protocols, and at least 20 000 events were counted on a FACSAria II instrument (BD Biosciences). Data were then analyzed using the software program FlowJo.

### Quantification of TCR Rearrangement Excision Circles (TRECs) and Kappa-Deleting Element Recombination Circles (KRECs)

The RT-PCR reactions were performed in a volume of 20 µL, containing 2×TK PCR Master Mix, 0.96 µL TREC mix, 0.96 µL KREC mix, 0.9 µL RNase P Mix, 0.4 µL 5× ROX (all from NuProbe, China), and 5 µL DNA, 1.72 µL sterile double distilled water. The 96-well plate reactions were carried out on ABI 7500 real-time PCR systems (Applied Biosystems), with a heating cycle at 95°C for 10 minutes, followed by 45 cycles of 15 seconds at 95°C and 60 seconds at 60°C. An individual cycle threshold for TRECs, KRECs, or RNase P was fixed for automated data collection and analysis of the amplification during the exponential phase. Calibration curves were generated by 10-fold serial dilution using TREC-KREC-RNase P construct containing plasmids. All analyzed RT-qPCR assays fulfilled the quality requirements of similar slopes and R2 values >0.99.

### Neutrophil Apoptosis Assay

Percentages of apoptotic neutrophils were determined using an Annexin V-FITC apoptosis detection kit (eBioscience). Neutrophils (1 × 10^6^/mL in 1 mL aliquots) were incubated (37°C, 5% CO_2_) with Lipopolysaccharide (LPS; Sigma) and FAS (Biolegend) for 16 h, and after the desired culture period, unstimulated neutrophils were washed once in room-temperature phosphate buffered saline (PBS) containing 10% fetal bovine serum, and incubated for 10 min with FITC-conjugated Annexin V and 7-AAD at room-temperature. Cells were then counterstained with propidium iodide and analyzed on a FACS Canto instrument (BD Biosciences).

### Respiratory Burst Assays

Respiratory burst was detected using a dihydrorhodamine (DHR)-1,2,3 flow cytometry assay. In the presence of peroxidase or an equivalent catalyst, H_2_O_2_ oxidizes DHR to rhodamine, which emits fluorescence when stimulated. For each blood sample from patients and HCs, two reactions were prepared as follows: in one tube, 50 μL blood was incubated with 20 μL of 2 ng/L phorbol 12-myristate-13-acetate (PMA; Sigma-Aldrich) and 6 μL 40 µmol/L DHR (Sigma-Aldrich); in another tube, 50 μL blood was incubated with 20 μL PBS and 6 μL 40 mol/L DHR. All reactions were incubated for 20 min at 37°C. After incubation, erythrocytes in the samples were lysed by incubation in lysing solution for 15 min. Following centrifugation (2000 rpm/5 min, room temperature) and washing with PBS, cells were examined using a FACS Canto II flow cytometer (BD Biosciences), and data were analyzed using FACS Diva software. The results are based on the stimulation index (SI = geometric mean of fluorescence intensity of PMA – incubated neutrophils/geometric mean of fluorescence intensity of PBS-incubated neutrophils).

### Quantification of NETs by Plate Assay

NETs formation was induced essentially as described previously (14). Neutrophils from patients and HC were incubated in triplicate with or without PMA stimulation, in black, 96-well plates for the scheduled period of time. Neutrophils were stained with Sytox Green (2.5 μM) for 5 min, 30 min, 1 h, 2 h, 3 h, and 4 h, and fluorescence (excitation, 485 nm; emission, 520 nm) was measured using a POLARstar fluorescence plate reader (BMG Technologies, Germany), according to the method of Halla et al (15). The fluorescence of neutrophils was detected at each time-point, and mean values were calculated for each time-point.

### Data Analysis

Data analysis was performed using GraphPad Prism 7.0a (Graphpad Software, USA), except for flow cytometry data, which were analyzed using FlowJo 10.3 (TreeStar, USA).

## Results

### Clinical Phenotypes and Laboratory Data

Two boys from two unrelated families were included in this study; patients 1 and 2 were 8 and 11 years old at the time of writing this report, respectively. Neutropenia was first documented in patient 1 when he was hospitalized with pneumonia at 3 months old. He was diagnosed with G6PC3 deficiency and received G-CSF treatment from age 3. G-CSF therapy was stopped when he was 7 years old. He was also administered oral trimethoprim-sulfamethoxazole as infection prophylaxis. In patient 2, neutropenia was first noted at 4 years old, during a physical examination. He was diagnosed with G6PC3 deficiency at age 8 and received regular G-CSF treatment from age 10. He has not received trimethoprim-sulfamethoxazole prophylaxis. Vaccinations were up-to-date in both patients, without any reported complications. Educationally, both patients were at an age-appropriate level.

Both patients had histories of pneumonia, inguinal hernia, cryptorchidism, and recurrent oral ulcers. However, their infections were not severe. Patient 1 also had asthma and otitis media. Patient 2 had prominent ectatic superficial veins on his limbs and presented with abdominal pain at age 6, was diagnosed with IBD by colonoscopy, and the abdominal pain symptoms were effectively relieved by long-term oral thalidomide treatment. Both patients had initial mild malnutrition, but their height and weight gradually returned to expected levels as their neutrophil counts stabilized (**Table 1**). Patient 1 had suffered recurrent upper respiratory tract infection and otitis media after discontinuation of G-CSF treatment, and patient 2 had not experienced recent severe infections.

**Table 1:** Clinical characteristics of patients with G6PC3 mutations.

	Haematological features	Infections	Urogenital abnormalities	Cutaneous abnormalities	Digestive tract	Vascular and Cardiac features	Growth and Development	Other features	Treatment
P1	Neutropenia, Anemia, Intermittent thrombocytopenia	Recurrent Pneumonia, Recurrent ulcer, Otitis media	Cryptorchidism, Bilateral inguinal hernia	None	None	None	Short stature	Asthma	Received G –CSF treatment for 4 years, discontinued for half year, Rrimethoprim-sulfamethoxazole
P2	Neutropenia	Recurrent Pneumonia, Recurrent ulcer	Cryptorchidism, Bilateral inguinal hernia	Prominent superficial venous pattern	IBD	None	Short stature	Slight scoliosis	During G-CSF treatment

**Figure 1:**
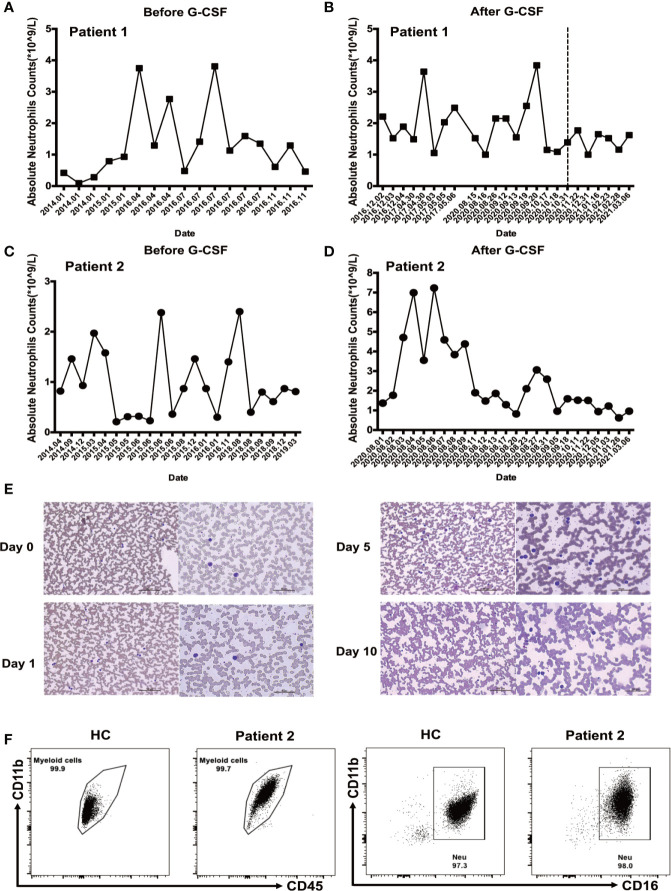
Neutrophil counts before and after granulocyte colony-stimulating factor (G-CSF) treatment. **(A)** Neutrophil count in patient 1 before G-CSF treatment. **(B)** Neutrophil count during G-CSF treatment in patient 1; Measurements after discontinuation of G-CSF treatment are shown on the right side of the dotted line. **(C)** Neutrophil count before G-CSF treatment in patient 2. **(D)** Neutrophil count during G-CSF treatment in patient 2. **(E)** Images of peripheral blood samples from patient 2 before and during G-CSF treatment, stained with Wright’s stain. **(F)** Neutrophils dominate Ficoll-Paque enriched SCN4 granulocyte fractions. Flow cytometry analysis was performed on Ficoll-Paque enriched granulocytes from patient 2 and healthy control to examine the number of neutrophils. The percentages of myeloid cells (CD11b+CD45+CD16+) and neutrophils (CD11b+CD45+CD16+) were determined.

Laboratory data revealed baseline absolute neutrophil counts (ANC) < 1 × 10^9^/L, which were noncyclic and transiently reversible by infection in both patients before G-CSF treatment (**Figures 1A, C**), and > 1 × 10^9^/L following G-CSF treatment (**Figures 1B, D**). ANC has remained > 1 × 10^9^/L for 6 months following G-CSF treatment suspension in patient 1 (**Figure 1B**). Hematoxylin and eosin staining and microscopic examination of blood slides from patient 2 during initial G-CSF treatment showed that peripheral blood granulocytes gradually changed from mononuclear to multinucleated, and granulocyte count stabilized (**Figure 1E**). We have reported previously that the granulocyte population of SCN1 patients with *ELANE* mutation is dominated by eosinophils (16). In the present study, we isolated granulocytes from patient 2 and stained the cells with a series of mAbs. Then we used CD45+CD11b+CD16+ to recognize neutrophils. The results suggested that, unlike SCN1 patients, neutrophils are the dominating granulocytes in the blood of SCN4 patients (**Figure 1F**).

Immunological investigations were also performed (**Table 2**). Lymphocytic classification and immunoglobulin levels were consistent in both patients. Circulating CD3^+^CD8^+^, CD19^+^, CD56^+^ NK cell count was normal, whereas CD4^+^ T cell frequency and absolute number were slightly decreased; the predominant reduction was of naive CD4^+^ T cells. Both patients had normal immunoglobulin levels, and proliferation rates of CD4^+^ T cells, CD8^+^ T cells, and CD19^+^ B cells did not significantly differ from those of healthy donors on PMA stimulation. Neutrophils pathogen phagocytosis was normal in G6PC3-defective patients according to the result of the nitroblue tetrazolium assay.

**Table 2:** Immunologic features of lymphocytes and immunoglobulin in the patients.

	CD3%	CD8%	Naïve CD8+ T cells%	CD4%	Naïve CD4+ T cells%	NK%	CD19%	Naïve B cells%	CD4+T cells proliferation	IgG	IgA	IgM	IgE
P1	62.1%	38.02%^*^	11.6%	11.65%^#^	4.9%^#^	29.67%^*^	8.22%^#^	2.7%^#^	Normal	10.6g/L	0.135g/L	2.36g/L	9IU/ml
P2	61.31%	28.45%	60.6%	25.67%^#^	18.3%^#^	11.5%	27.17%	66.5%	Normal	25.4g/L	0.845g/L	1.35g/L	8.9IU/ml

^*^Increased, ^#^Decreased.

Bone marrow cytology was performed twice for both patients and revealed no difference in the proportion of protogranulocytes in bone marrow between G6PC3 patients and HCs; however, some of the cells were swollen and contained vacuoles with toxic particles.

### TCR and BCR Rearrangements, Thymic and Bone Marrow Outputs of T and B Lymphocytes Were Impaired in Patients With SCN4

To estimate TCR and BCR rearrangements, thymic and bone marrow outputs, TRECs and KRECs from two patients were analyzed. The copies of TRECs from the two patients and KRECs from patient 1 were significantly lower than in age matched HCs. The results indicate that V(D)J rearrangement and thymic output were impaired in both patients. But BCR rearrangement and bone marrow outputs were impaired only in patient 1 (**Table 3**).

**Table 3:** Quantification of TRECs and KRECs.

Patients	TRECs (copies/10^5cells)	KRECs (copies/10^5cells)
Healthy Control 1	3914	2115
Healthy Control 2	1490	1716
Healthy Control 3	2829	1172
Patient 1	129	6
Patient 2	142	1408

### Determination of G6PC3 Deficiency

**Figure 2:**
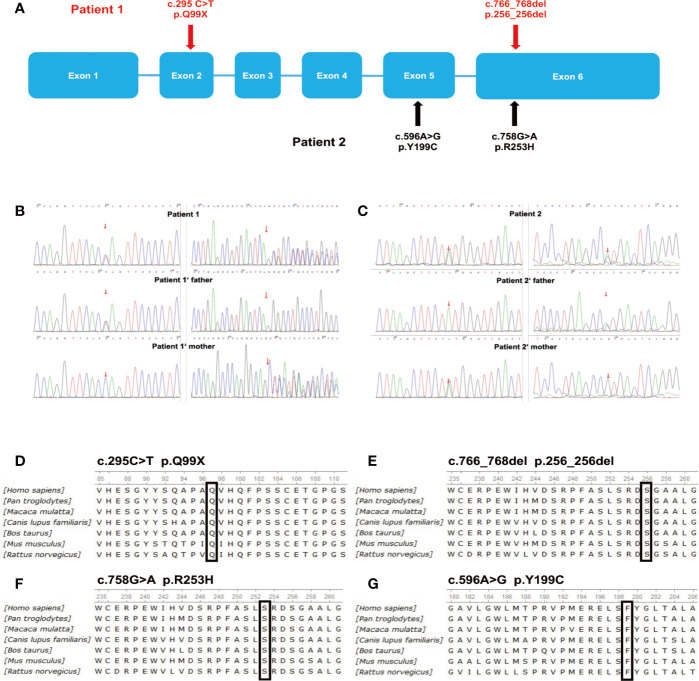
Deletions and point mutations detected by targeted deep sequencing. **(A)** Schematic representation of *G6PC3* gene mutations in the two patients. **(B)** Sequence analysis of *G6PC3* in patient 1, his father, and his mother. **(C)** Sequence analysis of *G6PC3* in patient 2, his father, and his mother. ClustalW alignment of human, chimpanzee, macaque, dog, cow, mouse, and rat G6PC3 protein sequences and mutations detected in patients 1 **(D, E)** and 2 **(F, G)**. **(D)** amino acids 85–111, the conserved glutamine at position 99 is highlighted in a black box; **(E)** amino acids 235–261, the conserved methionine at position 256 is highlighted in a black box; **(F)** amino acids 235–261, the conserved arginine at position 253 is highlighted in a black box; **(G)** amino acids 180–206, the conserved tyrosine at position 199 is highlighted in a black box.

Targeted deep sequencing was conducted to determine if the patients had genetic alterations in *G6PC3*. Mutations were detected in both patients and their family members (**Table 4**, **Figures 2A–C**). Our group has reported the mutations in Patient 1 previously (16), which were the heterozygous mutations in exons 2 and 6: a heterozygous missense mutation (c.295C>T, p.Q99X) in exon 2 inherited from his father, and a heterozygous deletion (c.766_768del, p.256_256del) in exon 6 inherited from his mother (**Figure 2B**), Patient 2 had heterozygous mutations in exons 5 and 6: his father carried a heterozygous missense mutation (c.758G>A, p.R253H) in exon 6, and his mother carried a heterozygous missense mutation (c.596A>G, p. Y199C) in exon 5 (**Figure 2C**). Sanger sequencing of genomic DNA was also conducted in both patients and their family members, and confirmed the mutations discovered by targeted deep sequencing. The missense mutation, c.758G>A (p.R253H) in patient 2 has been reported previously (17), while the other three mutations found in our two patients have not been previously reported in SNP databases and were not observed in 1000 control subjects. Separating intolerant from tolerant (SIFT) analysis shows that these residues are highly conserved among species (**Figures 2D–G**).

**Table 4:** G6PC3 gene mutation status in patients and their parents.

Patients	Exon	Mutation	Protein	Inherit
Patient 1	Exon2	c.295C>T	p.Q99X	Father
	Exon6	c.766_768del	p.256_256del	Mother
Patient 2	Exon 6	c. 758G>A	p.R253H	Father
	Exon 5	c.596A>G	p.Y199C	Mother

### Increased CXCR4 Expression on Neutrophils and NK Cells in Patients With SCN4

**Figure 3:**
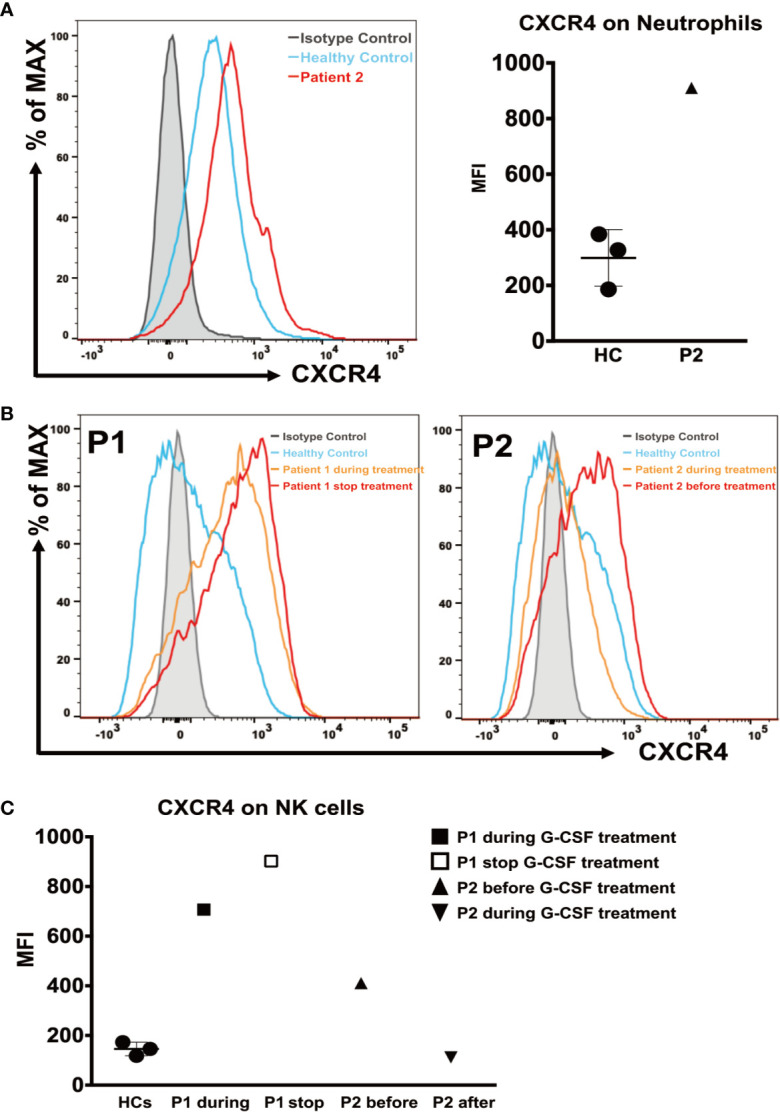
CXCR4 expression on neutrophils and NK cells. **(A)** (Left) Representative histograms of neutrophils from patient 2 during G-CSF treatment and healthy controls. (Right) The mean fluorescence intensity (MFI) of CXCR4 in neutrophils from patient 2 and healthy controls (n=3). **(B)** Representative histograms of NK cells from patient 1 during and after discontinuation of G-CSF treatment (Left), patient 2 before and during G-CSF treatment (Right), and healthy controls. **(C)** The MFI of CXCR4 on NK cells from patient 1 after discontinuation of G-CSF treatment, patient 2 before and during G-CSF treatment and healthy controls (n=3).

We analyzed CXCR4 expression on neutrophils from patient 2, and PBMCs from both patients, measured as the mean fluorescence intensity (MFI). The CXCR4 expression on neutrophils from patient 2 (MFI = 910) was higher during G-CSF treatment than in the HC samples (MFI = 299 ± 101.9) (**Figure 3A**). Patient 1 showed a dramatic increase in CXCR4 expression on CD56^+^NK cells during G-CSF treatment (MFI = 707) compared with HC samples (MFI = 145.7 ± 27.5), and the increase was more significant after he stopped G-CSF treatment (MFI = 902). Similarly, the MFI of CXCR4 expression on CD56^+^NK cells was also increased in patient 2 before he received G-CSF treatment (MFI =411), and almost returned to normal levels after G-CSF treatment (MFI =112) (**Figures 3B, C**). In contrast, CXCR4 expression in any of the other leukocyte subsets (CD4, CD8, and CD19) of SCN4 patients did not differ from that in HCs (data not shown).

### Neutrophil Respiratory Burst Was Impaired in Patients With SCN4 During G-CSF Treatment

**Figure 4:**
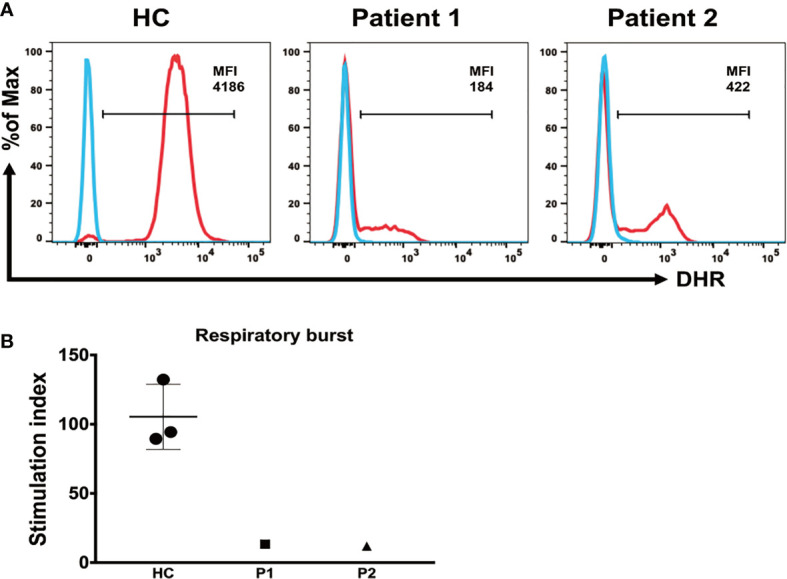
Detection of respiratory burst activity induced by PMA stimulation. **(A)** Whole blood was incubated in the absence or presence of PMA, as indicated. Respiratory burst was detected *via* conversion of DHR-123 in neutrophils. Median fluorescence intensity of the entire neutrophil-gated cell population, enclosed by the marker bar, is specified in each plot. **(B)** Mean (± SD) respiratory burst stimulation index (SI) values in healthy controls and patients 1 and 2.

To test the ability of neutrophils to generate and release ROS, we compared neutrophil respiratory burst capacity in patients and HCs. The respiratory burst capacity of stimulated neutrophils in both patients was low, and ROS generation and release in response to PMA were clearly diminished in patient 1 after discontinuation of G-CSF treatment and in patient 2 during G-CSF therapy, compared with HC samples (**Figure 4A**). The SI values in patients 1 and 2 were 13.31 and 11.92, respectively, which were significantly lower than those in HC samples (SI = 105.4 ± 23.42) (**Figure 4B**).

### Increased Neutrophil Apoptosis in Patients With SCN4

**Figure 5:**
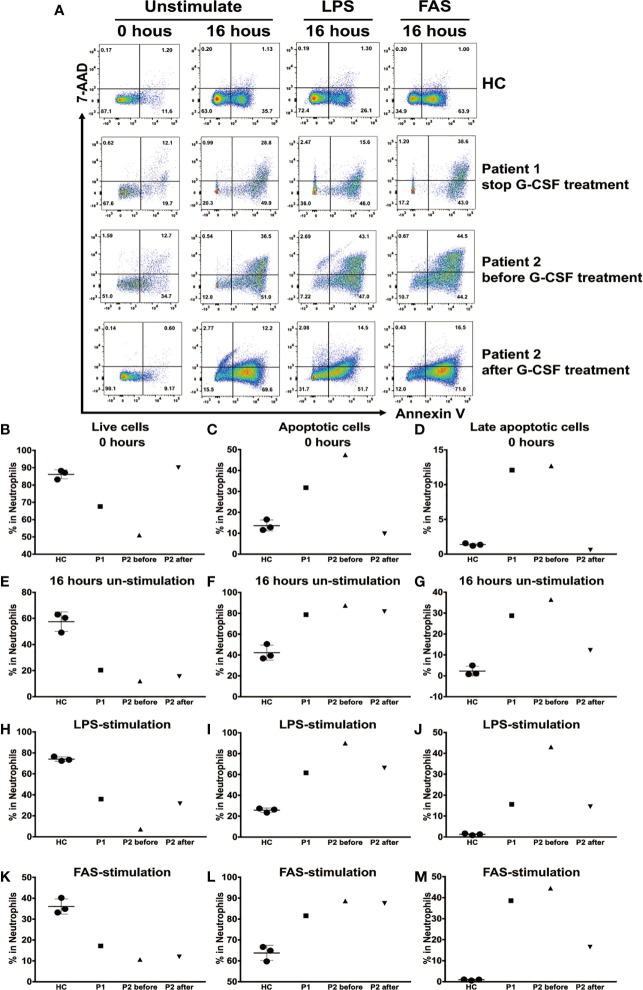
Apoptosis of human neutrophils analyzed using annexin V and propidium iodide staining. **(A)** Neutrophils from healthy controls (HCs) and patient 1 following cessation of granulocyte colony-stimulating factor (G-CSF) treatment, and patient 2 before and during G-CSF treatment, at baseline and following culture with or without lipopolysaccharide (LPS) or FAS for 16 h. Cells were stained for annexin V and propidium iodide, analyzed using flow cytometry. Mean (± SD) percentages of live neutrophils **(B)**, total apoptotic neutrophils **(C)**, and late apoptotic neutrophils **(D)** among total neutrophils at baseline. Mean (± SD) percentages of live neutrophils **(E)**, total apoptotic neutrophils **(F)**, and late apoptotic neutrophils **(G)** among total neutrophils after culture without stimulation for 16 h. Mean (± SD) percentages of live neutrophils **(H)**, total apoptotic neutrophils **(I)**, and late apoptotic neutrophils **(J)** among total neutrophils after culture with LPS for 16 h. Mean (± SD) percentages of live neutrophils **(K)**, total apoptotic neutrophils **(L)**, and late apoptotic neutrophils **(M)** among total neutrophils after culture with FAS for 16 h.

Next, we compared the apoptosis rates of neutrophils isolated from HCs, patient 1 after discontinuation of G-CSF treatment, and patient 2 before and during G-CSF therapy. Annexin V-positive cells were gated as apoptotic cells, and annexin V and propidium iodide double-positive cells as late apoptotic cells. To examine the spontaneous apoptosis of neutrophils, we detected the percentage of live cells, total apoptotic cells, and late apoptotic cells among total neutrophils at baseline and after incubation without stimulation for 16 h. In addition, we analyzed the percentages of apoptotic cells following incubation with LPS or FAS for 16 h, to determine apoptosis rates in activated neutrophils and neutrophils after induction of apoptosis, respectively (**Figure 5A**).

The percentages of live cells in patient 1 (67.6%) and patient 2 before G-CSF treatment (51%) were significantly lower than those in HCs (86.17% ± 2.63%) at baseline; however, the results in cells from patient 2 during G-CSF treatment (90.1%) were similar to those in HCs. By contrast, the percentage of apoptotic cells and late apoptotic cells in patients 1 and 2 before G-CSF treatment were significantly higher than those in HCs at baseline, while those in patient 2 after treatment were similar to HCs (**Figures 5B–D**).

The percentages of live cells in patients 1 and 2 before and during G-CSF treatment were lower, and that of total apoptotic cells higher, than those in HCs after 16 h incubation without stimulation (**Figures 5E–G**); however, the percentage of late apoptotic cells in patient 2 during G-CSF treatment was similar to that in HCs.

After incubation with LPS or FAS for 16 h, the percentages of live cells were lower, and those of total apoptotic cells higher, in patients 1 and 2 before and during G-CSF treatment, than those in HCs. Furthermore, following stimulation with LPS, the percentages of late apoptotic cells were slightly higher in patients 1 and 2 during G-CSF treatment and significantly higher in patient 2 before G-CSF treatment (**Figures 5H–J**). Nevertheless, following FAS stimulation, the percentage of late apoptotic cells was significantly higher in patient 1, while in patient 2 during G-CSF treatment, the percentage was only slightly higher (**Figures 5K–M**).

### Neutrophils From SCN4 Patients Could Not Release NETs

**Figure 6:**
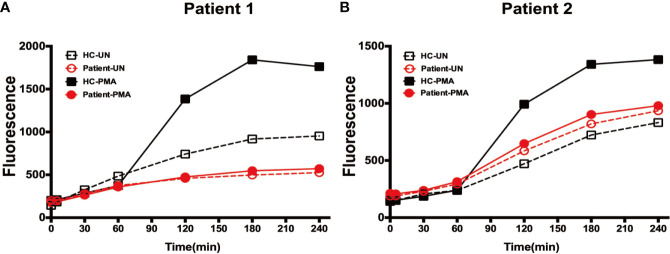
Expulsion of NETs from neutrophils. **(A)** Fluorescence generated by neutrophils from patient 1 and healthy controls at each time-point following incubation in the presence or absence of PMA stimulation. **(B)** Fluorescence generated by neutrophils from patient 2 and healthy controls at each time-point following incubation in the presence or absence of PMA stimulation.

Neutrophils treated with PMA form NETs, as stimulation with PMA triggers the assembly and activation of NADPH oxidase, which is essential for NETs formation. We used the Sytox Green assay, which evaluates the amounts of extracellular DNA, to measure NETs formation. Sytox Green fluorescence was observed for 240 min, which is the time taken for neutrophils to form NETs following PMA stimulation (15). Analysis of neutrophils from both patients during G-CSF therapy and HC using this system demonstrated that NETs formation was significantly defective in neutrophils from both patients (**Figures 6A, B**).

## Discussion

Here, we described two Chinese SCN4 patients with *G6PC3* mutations. Both patients presented with neutropenia and some typical clinical characteristics of SCN4. Both patients received G-CSF treatment, while G-CSF therapy has been stopped in patient 1 for 6 months.

In this study, we report one de nove *G6PC3* mutation site in patient 2. The missense mutation, c.596A>G (p.Y199C), has not been detected in any other patient. The other missense mutation, c.758G>A (p.R253H), is common in patients with G6PC3 deficiency of Middle Eastern descent (17). The compound heterozygous mutations in patient 1 have been reported by our group previously. Sequencing of DNA from the parents of both patients demonstrated that the mutations were bi-allelic, and both paternally and maternally inherited. All of the amino acid residues affected by mutations detected in this study are highly conserved across species. At present, the relationship between genotype and clinical phenotype of the disease is unclear. Mild clinical symptoms in both patients may be related to the compound heterozygous mutations.

The number of naive CD4^+^ T cells was lower in our two patients, which is consistent with previous reports (18). Previous studies have reported that some patients with the G6PC3 mutation present with thymic hypoplasia (19, 20). To investigate the possible reasons, we measured the number of TRECs and T cell proliferation. The results showed that the number of TRECs was lower in both patients, but CD4^+^ T cell proliferation in response to PHA was normal (data not shown). Chest X-ray scans revealed no evidence for an absent thymus gland or a reduction in its volume. Together, these results suggested that the two patients also had partial thymic dysplasia.

Unlike T cells, the level of naive CD19^+^ B cells and the number of KRECs were decreased only in patient 1, but the classification and quantities of immunoglobulin were normal. These results suggested that G6PC3 deficiency may impair bone marrow output and BCR rearrangement in some patients.

G-CSF treatment of G6PC3 defective mice corrects neutropenia, improves neutrophil functions, stimulates neutrophil glucose uptake, and improves energy homeostasis and functionality (8). G-CSF can delay neutrophil apoptosis by modulating apoptotic mediators but cannot prevent their accelerated death (5, 8, 21, 22). Neutrophils have a short half-life in blood in the absence of pro-survival signals (23), and will survive for approximately 18–24 h before undergoing constitutive apoptosis in culture (24, 25). FAS is involved in the extrinsic pathway of neutrophil apoptosis and expressed on the surface of neutrophils. Ligation of death receptors by their ligands such as FAS-associated protein with a death domain, promotes the recruitment of adaptor molecules and the aggregation and activation of caspase-8, leading to apoptosis (26, 27). Further, LPS-induced inflammation can diminish sensitivity to FAS stimulation and rescue the apoptosis reaction.

Myelokathexis has been discussed as a mechanism of neutropenia in cases of G6PC3 deficiency associated with increased expression of the bone marrow homing receptor CXCR4 which could expressed selectively on neutrophils and NK cells. McDermott et al. have showed that the expression of CXCR4 is significantly higher in neutrophils and NK cells from patients with G6PC3 deficiency than in those from HCs, and declines as the G-CSF dose increases (9). It is known that interfering with the CXCR4-CXCL12 receptor-ligand interaction is one of the mechanisms by which G-CSF corrects neutropenia (28). Our data are consistent with those of a previous report. In the patient 1, the increase in CXCR4 expression are more dramatic after discontinuation of G-CSF treatment than during treatment. This result suggests that the CXCR4 levels may rise again after discontinuation of G-CSF treatment in SCN4 patients. However, the increase of CXCR4 expression was not consistent between the two patients, which may be related to individual differences.

Our data show that, at baseline, proportions of live, total apoptotic, and late apoptotic neutrophils in a patient treated with G-CSF were similar to those of HCs, while the neutrophils in patients before G-CSF treatment or after stopping treatment showed significant spontaneous apoptosis; however, the number of apoptotic neutrophils remained significantly increased in patients after 16 h of *in vitro* culture without stimulation, and in controls these were primarily early apoptotic neutrophils, while samples from patients not undergoing G-CSF treatment contained mainly late apoptotic neutrophils. The total number of apoptotic neutrophils was clearly increased before and during G-CSF treatment in patients with mutated *G6PC3*; however, the number of late apoptotic neutrophils decreased somewhat during G-CSF treatment, relative to before or after cessation of treatment, after 16 h of *in vitro* culture with FAS. By contrast, total apoptotic cells, particularly late apoptotic neutrophils, remained significantly higher in samples from patients before G-CSF therapy, following 16 h of LPS stimulation. Nevertheless, total apoptotic neutrophils, and even late apoptotic neutrophils, were lower following G-CSF treatment and after cessation of G-CSF treatment, relative to patients before G-CSF therapy, but were still higher than those in HCs. Together, these data indicate that neutrophils from patients with *G6PC3* mutation were mainly in late apoptosis at baseline or after FAS induction, while in HCs they were mainly in early apoptosis after FAS induction. GM-CSF could suppress FAS-induced apoptosis of neutrophils by inhibiting FADD binding to FAS, through redundant actions of PI-3K and MEK1-ERK1/2 pathways downstream of classical PKC (29). Neutrophils apoptosis showed significant improvement at FAS-induced state in our patient after G-CSF treatment. The results confirm that G6PC3 deficiency leads to accelerated neutrophil apoptosis, and that G-CSF can delay apoptosis, consistent with previous findings from G6PC3 defective mice (8).

Interestingly, the total number of apoptotic neutrophils in patients who discontinued G-CSF treatment was reduced compared with that in patients before G-CSF treatment; however, late apoptotic neutrophil numbers were similar to those before treatment, which may explain why neutrophil counts remained at approximately 1 × 10^9^/L after G-CSF treatment was discontinued, as early apoptosis was reduced. Since spontaneous apoptosis after 16 h and FAS-induced apoptosis of neutrophils from the patient who discontinued G-CSF treatment were similar to those before G-CSF treatment, we conclude that the apoptotic state of neutrophils did not differ before and after discontinuation of G-CSF treatment.

We also tested neutrophil respiratory burst, pathogen phagocytosis, and NETs expulsion functions in patients after G-CSF treatment. The results of our nitroblue tetrazolium assay showed that neutrophil pathogen phagocytosis was normal in G6PC3-defective patients, while respiratory burst and ROS production were dysfunctional, and NETs production was deficient.

Neutrophils are known to generate and release large amounts of ROS *via* the NADPH oxidase system, allowing these cells to respond to various stimuli with a respiratory burst. Previous data have demonstrated that G-CSF treatment could improve respiratory burst, chemotaxis, and calcium flux activities in neutrophils from G6PC3^-/-^ mice (8); however, our data showed that respiratory burst dysfunction continued in patients with G6PC3 deficiency following G-CSF treatment.

NETs are produced by activated neutrophils and comprise large, extracellular, fibrous networks, composed of cytosolic and granule proteins assembled on a scaffold of decondensed chromatin. NETs function as physical and antimicrobial barriers that first extracellularly confine and then kill pathogens at sites of inflammation. NETs can trap, neutralize, and kill bacteria, fungi, viruses, and parasites, and prevent bacterial and fungal dissemination, and their formation and release occur primarily through NETosis (30, 31), a specific type of cell death that differs from both necrosis and apoptosis and requires ROS production (31), and in which neutrophils arrest their actin dynamics and depolarize after activation. Next, the nuclear envelope disassembles, and nuclear chromatin decondenses into the cytoplasm of intact cells, mixing with cytoplasmic and granule components. The plasma membrane then permeabilizes, and NETs expand into the extracellular space 3–8 h after neutrophil activation (31, 32). Activated neutrophils generate and release large amounts of ROS *via* the NADPH oxidase system, and this process is pivotal for NETosis (33, 34). Meanwhile, Formation of NETs has an important relevance with glycolysis (28).

It is known that G6Pase-deficiency can impair glycolysis and hexose monophosphate shunt activities, leading to reductions in nicotinamide adenine dinucleotide phosphate (NADPH) oxidase activity, and glycolysis was impaired in patients with G6PC3 deficiency (10, 28). Our results also confirmed that respiratory burst was impaired in patients with G6PC3 mutation. As mentioned above, NETosis involves programmed neutrophil death; however, deficiency of glycolysis and ROS production in patients with G6PC3 deficiency leads to increased neutrophil apoptosis and inability to undergo effective programmed death. This may account for the deficiency in NETs formation in neutrophils from SCN4 patients.

Studies in patients with cancer have shown that neutrophils may be more sensitive to formation of NETs in the presence of G-CSF (35). However, SCN4 patients treated with G-CSF exhibit deficient NETs production. We considered that G-CSF can delay neutrophil apoptosis but not reduce it; hence, neutrophils also cannot produce NETs in patients with G6PC3 deficiency treated with G-CSF treatment. Therefore, despite near-normal neutrophil counts, patients with G6PC3 deficiency remain at risk of infection.

In summary, two patients were diagnosed with SCN4 with G6PC3 deficiency, and their clinical features and immunology were described. We detected limited thymus output and TCR rearrangement in SCN4 patients, and bone marrow output and BCR rearrangement in some patients were damaged. G-CSF is a main treatment for SCN4 patients, and we compared neutrophil functions in patients at different treatment time-points. Our results indicate that spontaneous and FAS-induced neutrophil apoptosis were significantly increased in patients without, or after discontinued, G-CSF treatment, and improved only slightly in response to G-CSF treatment. While G-CSF treatment may also partially ameliorate neutropenia in patients with G6PC3 deficiency by decreasing CXCR4 expression. Further, neutrophil respiratory burst and NETs production remained impaired in patients with G6PC3 deficiency following G-CSF treatment.

These data suggest that G-CSF treatment is insufficient for patients with G6PC3 deficiency, who remain at risk of infection. However, untreated G6PC3 deficiency can be fatal. The oldest patient ever described with G6PC3 deficiency who was non-compliant with treatment died at 37 years old due to infective endocarditis (20). The youngest reported age of death in a patient with G6PC3 deficiency was at 9 months due to severe lung infection (36), demonstrating that the natural course of G6PC3 deficiency can be fatal. Where possible, regular G-CSF treatment and long-term prevention of infection are the best therapies for patients with SCN4. G6PC3 neutropenia may also be associated with myelodysplasia and AML. Lifelong G-CSF therapy is required but patients are at a risk of developing leukemia (37). Thus, it is important to monitor closely for signs of leukemia in SCN4 patients. Once leukemia occurs in SCN4 patients, hematopoietic stem cell transplantation is the most important choice of treatment (38). Hoffmann D et al. have reported that lentiviral genetic correction of SCN iPSCs with a codon-optimized G6PC3 transgene restores granulopoiesis and reduces apoptosis of *in vitro* differentiated myeloid cells (39). This may provide another therapeutic option.

## Data Availability Statement

The original contributions presented in the study are included in the article. Further inquiries can be directed to the corresponding authors.

## Ethics Statement

The studies involving human participants were reviewed and approved by Ethics Committee of the Children’s Hospital of ChongQing Medical University. Written informed consent to participate in this study was provided by the participants’ legal guardian/next of kin. Written informed consent was obtained from the individual(s), and minor(s)’ legal guardian/next of kin, for the publication of any potentially identifiable images or data included in this article.

## Author Contributions

RD performed research, analyzed data, and wrote the paper; GL performed research and analyzed data; WL performed research and analyzed data and reviewed and revised the manuscript; WT, JC, QL, LY, MZ, ZT and LZ provided help in performing the research; XY, YW, YD, YA, ZZ and XT helped obtain clinical samples; XZ conceptualized and designed the study and reviewed and revised the manuscript; and all authors approved the final manuscript as submitted and agree to be accountable for all aspects of the work.

## Funding

This work was supported by the National Natural Science Foundation of China (81801637, 81620108014).

## Conflict of Interest

The authors declare that the research was conducted in the absence of any commercial or financial relationships that could be construed as a potential conflict of interest.
